# Bone Regeneration and Soft Tissue Enhancement Around Zygomatic Implants: Retrospective Case Series

**DOI:** 10.3390/ma13071577

**Published:** 2020-03-29

**Authors:** Miguel Peñarrocha-Diago, Juan Carlos Bernabeu-Mira, Alberto Fernández-Ruíz, Carlos Aparicio, David Peñarrocha-Oltra

**Affiliations:** 1 Stomatology Department, University of Valencia, 46010 Valencia, Spain; miguel.penarrocha@uv.es (M.P.-D.); direccion@clinicafernandez.es (A.F.-R.); david.penarrocha@uv.es (D.P.-O.); 2 Private Practice, 07800 Ibiza, Balearic Islands, Spain; 3 Hepler Bone Clinic, ZAGA Center, 08017 Barcelona, Spain; carlos.pisanlof@gmail.com

**Keywords:** zygomatic implants, zygomatic implant complications, bone regeneration, soft tissue regeneration

## Abstract

Purpose: To present a case series of zygomatic implants combined with bone regeneration and soft tissue enhancement techniques to reduce the risk of biological delayed complications such as maxillary sinusitis and soft tissue recession. Materials and methods: Zygomatic implants placed simultaneously with different bone regeneration techniques (buccal, palatal and buccal-palatal bone regeneration) and soft tissue enhancement techniques (pediculate and free connective tissue graft) were followed for at least 12 months. The following information was collected: patient age and sex, number of zygomatic implants, zygomatic implant success rate, zygomatic implant position according to classification of the Zygomatic Anatomy Guide Approach (ZAGA), sinus membrane perforation, type and outcome of the bone regeneration or the soft tissue enhancement technique, bone gain (width and length along the zygomatic implant) and keratinized buccal mucosa width, duration of follow-up, loading protocol (immediate or delayed) and biological complications (maxillary sinusitis and soft tissue recession). Results: Thirty-one zygomatic implants placed in 19 patients were included. All implants were successful and none of the implants presented biological complications. The bone regeneration technique was successful in 30 of 31 cases with a mean palatal bone width of 3 mm, buccal bone width of 2.65 mm, palatal bone length of 6.5 mm and buccal bone length of 8.3 mm. The success rate of soft tissue enhancement was 100% and it established at least 2 mm of keratinized buccal mucosa width in all implants. Conclusions: Within the limitations of the present study, bone regeneration and soft tissue enhancement techniques were useful to establish more favorable conditions of the peri-implant tissues around zygomatic implants. This could prevent biological complications such as maxillary sinusitis and soft tissue recessions. Prospective and randomized controlled clinical trials with longer follow-up periods are advisable.

## 1. Introduction

The original zygomatic implant placement technique was described by Branemark with a 97% of success rate in 81 treated patients [1]. The classical protocol introduced the placement of conventional implants in the maxillary anterior region in combination with posterior zygomatic implants through a palatal entrance and extensive sinus opening [1,2,3,4,5]. This method was proposed for the rehabilitation of atrophic maxillae (grade V and VI of Cawood–Howell classification [6]) without the use of grafts [1,2,3]. Zygomatic implant-supported fixed prostheses were found to be similar to conventional implant-supported fixed prostheses in terms of patient satisfaction [7].

Biological complications, such as maxillary sinusitis and buccal mucosal recession, have been reported [8]. The classical intrasinus position was associated with a maxillary sinus infection rate of 2.3–13.6% [8,9], due to oroantral communication after marginal peri-implantitis in thin palatal bone, pending micromovements of the zygomatic implant and severe atrophy of the maxillae.

Posteriorly, a number of authors described different modifications of the original Branemark technique, seeking to prevent biological complications and bulky prostheses. In the year 2000, Stella and Warner [5] introduced in a technical note the “slot technique”: a reduced slot-shaped opening in the sinus wall was proposed to visualize the implant path. Implant entrance to the sinus cavity was performed through the crest, allowing for a better prosthetic design with satisfactory results, in the presence of a concave maxillary wall and moderate-advanced atrophy [10,11]. Several authors [12,13,14,15] described a different approach for zygomatic surgery known as the exteriorized technique. In the presence of a concave maxillary wall, the implants are partially placed outside the maxillary sinus and covered just with soft tissue. This approach eliminates the need for maxillary “window antrostomy” or the creation of a slot previous to the surgery.

A comprehensive concept named Zygomatic Anatomically Guided Approach (ZAGA) has been proposed [16] and evaluated [17] with promising results. ZAGA is a classification focused on a variety of possibilities of implant trajectory from the intrasinus to an eventual extrasinus passage according to the patient anatomy variations.

On analyzing the evolution of the abovementioned techniques, it is seen that the position of the neck of the zygomatic implants has been mobilized laterally from an intrasinus position to an extrasinus position. Different anterior techniques [5,12,13,14] have thus sought to avoid maxillary sinus complications. However, hard and soft tissue infective and aesthetic complications (exposure of the implant threads [15]) were detected [18,19].

Regeneration of the lost peri-implant tissues around the coronal part of the zygomatic implants could be useful to ensure optimal implant prognosis and prevent the abovementioned complications due to a lack of osseointegration at the marginal level of the implant.

Peri-implant hard and soft tissues are crucial for avoiding complications around conventional implants. Two aspects have been studied in this regard: buccal cortical bone and the thickness and width of the peri-implant keratinized mucosa. On the one hand, a recent systematic review [20] has reported that a buccal cortical thickness of close to 2 mm was associated with less vertical bone resorption and less mucosal recession. On the other hand, the presence of sufficient keratinized mucosa thickness exerts a protective effect against marginal bone loss [21,22].

These abovementioned concepts to minimum necessary peri-implant tissues could be extrapolated to zygomatic implants. A number of techniques for bone and soft tissue enhancement around zygomatic implants have been described, with high success rates. Regarding bone regeneration, a sinus lift during the zygomatic implant placement has been proposed [23,24]. Regarding soft tissue enhancement, dissection of the buccal fat pad [25] and the ZAGA “Scarf Graft” (pediculate connective tissue grafting [26]) have been utilized to prevent buccal mucosal recession.

The present study describes a retrospective case series of zygomatic implants combined with simultaneous bone regeneration and soft tissue enhancement techniques to reduce the risk of biological complications such as maxillary sinusitis and soft tissue recession. The prespecified hypothesis is that regenerative techniques around the coronal part of zygomatic implants will be effective to prevent the abovementioned complications.

## 2. Material and Methods

A descriptive retrospective case series of treated patients with zygomatic implants in conjunction with bone regeneration and soft tissue enhancement techniques was performed. This descriptive study was written according Clinical Case Reporting Guideline (CARE) [27]. All subjects gave their informed consent for inclusion before they participated in the study. The study was conducted in accordance with the Declaration of Helsinki, and the protocol was approved by the Ethics Committee of the University of Valencia (UV-INV_ETICA-1263997). The database of the Oral Surgery Unit (Department of Stomatology, Faculty of Medicine and Dentistry, University of Valencia, Spain) was consulted to collect the information.

### 2.1. Patient Selection and Operating Procedure

All patients were treated by the same oral surgeon (MPD) between June of 2016 and November of 2019. At the first appointment, a complete anamnesis, oral exploration and radiographic study (extraoral panoramic radiograph and cone bean computed tomography (CBCT) (Planmeca Promax ^®^ 3D Max and 2D S3, Helsinki, Finland)) were made. The selection of the patients depending on criteria specified in Table 1.

#### 2.1.1. Indication for Regenerative or Enhancement Method

Different techniques of bone regeneration and improvement of peri-implant soft tissues have been indicated depending on the emergence of the implant and the state of the alveolar process after implant placement (Table 2). As a premise, it should be remembered that the coronal part of the implant has to obtain minimal peri-implant tissues [20,21,22].

#### 2.1.2. Bone Regeneration

**Figure 1:**
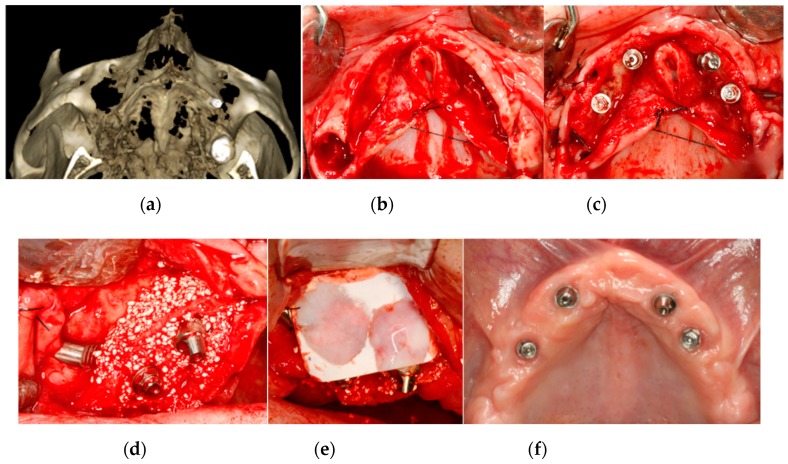

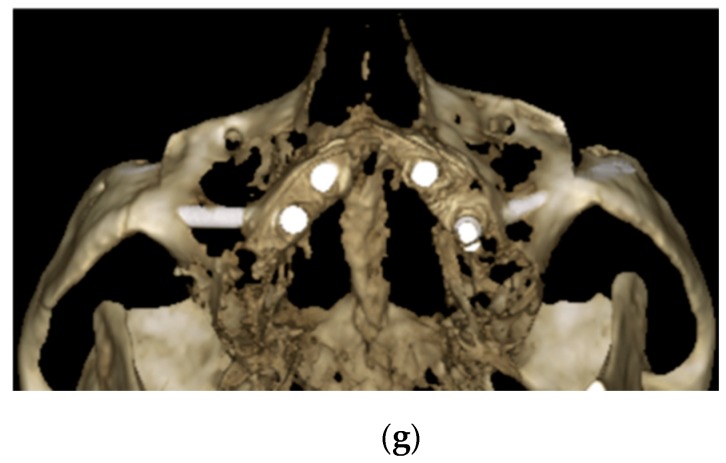
(**a**) Atrophic alveolar bone crest seen in a preoperative three-dimensional reconstruction CBCT image. (**b**) Atrophy of edentulous alveolar crest seen in an intraoral clinical image. (**c**) Placement of two posterior zygomatic implants (Branemark System Zygoma^®^, Nobel Biocare, Sweden). The coronal part of the zygomatic implant remains in a palatal position, and buccal fenestration is presented. (**d**) Buccal-centered image showing the buccal and palatal bone graft (KeraOs^®^, Keramat, Spain))**.** (**e**) Buccal-centered image showing overlap of the resorbable collagen membrane (Creos Xenoprotect^®^, Nobel Biocare) upon the bone graft. (**f**) Soft tissue healing at one month after implant placement. (**g**) Postoperative three-dimensional reconstruction CBCT image showing palatal and buccal bone regeneration around the implants at 6 months after surgery.

Peri-implant bone defects or thin buccal/palatal corticals were regenerated with a mixture of particulate synthetic bone graft (beta-phosphate tricalcium (KeraOs ^®^, Keramat, Spain)) with autogenous bone and resorbable collagen membranes (Creos Xenoprotect ^®^, Nobel Biocare, Sweden) fixed with surgical pins (Meisinger ^®^, Sanhigia, Spain). Management of the soft tissue without flap pressure was achieved through two-plane suturing (horizontal double and simple stitches). Bone regeneration was performed palatal, buccal or palatal and buccal. An example of buccal and palatal bone regeneration is illustrated in Figure 1.

#### 2.1.3. Buccal Soft Tissue Enhancement

**Figure 2:**
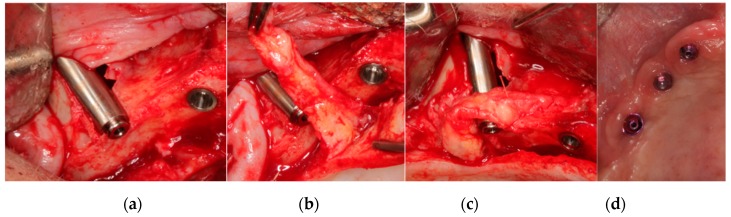
(**a**) Buccal bone dehiscence present in a zygomatic implant with a smooth neck surface (IPX-Tilted System, Smooth, Galimplant S.L., Sarria, Galicia, Spain). More than half of the implant diameter outside the alveolar bone crest. (**b**) Pediculate subepithelial tissue palatal rotation graft. (**c**) Mesial and distal fixation of the soft tissue graft through suture. (**d**) Clinical image of soft tissue healing at 6 months after implant placement.

**Figure 3:**
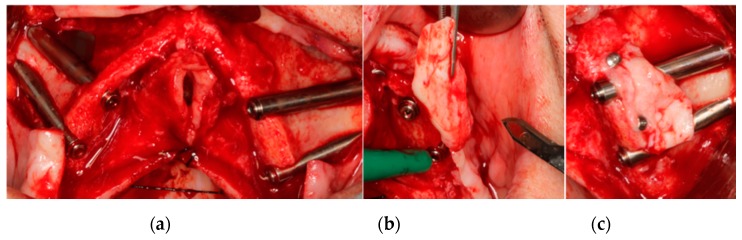

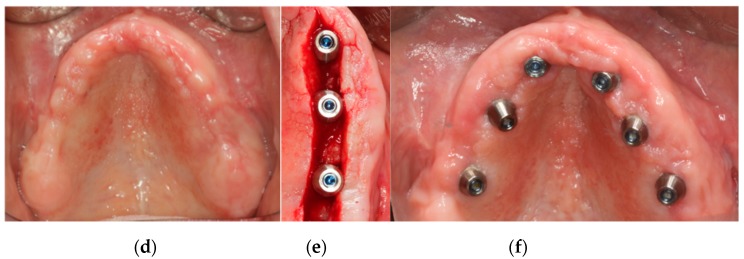
(**a**) Zygoma Quad system with buccal dehiscences seen in both quadrants (Smooth IPX-Tilted System^®^, Galimplant S.L, Sarria, Galicia, Spain). (**b**) A free connective soft tissue graft is collected from the palatal flap. (**c**) Soft tissue graft is repositioned with two surgical pins around the neck of the zygomatic implants. (**d**) Soft tissue healing at one month after the operation. (**e**) The gained soft tissue volume is seen in second stage surgery. (**f**) Soft tissue healing at three weeks after second stage surgery. The buccal mucosa is thick and keratinized.

Palatal rotated connective tissue flaps or free connective tissue grafts were performed to improve buccal soft tissues around the zygomatic implants. Palatal rotated flaps (Figure 2) were fixed buccally to the zygomatic implants using resorbable sutures through small perforations created in the bone alveolar crest mesial and distal of the zygomatic implant. Free connective tissue grafts (Figure 3) were collected from the palatal flap and fixed buccally around the coronal part of the zygomatic implants by two surgical pins.

#### 2.1.4. Immediate Loading

Immediate loading was performed when the zygomatic implants had primary stability (insertion torque at least 35 Ncm) and if demanded by the patient.

#### 2.1.5. Follow-up

All patients were checked after two weeks (suture removal), at 6 months (second-stage surgery), at 8 months (prosthetic loading) and each year after prosthetic loading. A CBCT scan was performed to evaluate the bone regeneration and the health of the maxillary sinusitis at the 6 months. Protheses removal and prophylaxis were conducted every year.

### 2.2. Data Gathering

The following information was collected in all cases: patient age and sex, number of zygomatic implants, sinus membrane perforation, zygomatic implant position according to ZAGA classification [16], zygomatic implant success, type and success rate of the bone regeneration or of the soft tissue enhancement technique, bone gain (width and length along the zygomatic implant) or keratinized buccal mucosa width, duration of follow-up, loading protocol (immediate or delayed) and biological complications (maxillary sinusitis and soft tissue recession).

The evaluation of maxillary sinusitis and soft tissue recession was made by radiological study and clinical exploration. For maxillary sinusitis diagnosis, the CBCT images were evaluated at 6 months to discard maxillary sinus occupation and the clinical exploration assessed possible symptoms and signs at each follow-up visit such as: Facial pain or pressure, facial congestion or fullness, nasal obstruction, purulent discharge, hyposmia or anosmia, purulence on examination and fever [28]. For soft tissue recession, a clinical visual exploration was performed, and defects were measured through a periodontal probe.

Bone regeneration success in cases with immediate loading was evaluated by CBCT images at 6 months. This evaluation was performed during the second-stage surgery in cases without immediate loading. Soft tissue enhancement success was based on the presence or absence of necrosis during the next two weeks after the implant placement.

**Figure 4:**
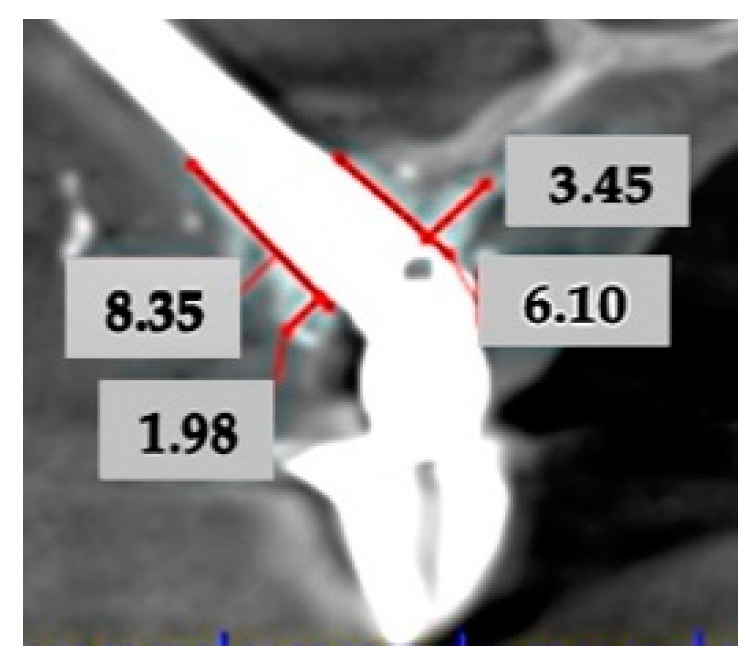
Bone gain measurement by CBCT at 6 months in two directions: through a perpendicular buccal and palatal lines to the implant axis (width) and through a parallel buccal and palatal lines to the implant axis at level of coronal part of the implant (length).

Bone gain was measured in the CBCT images at 6 months in two directions: through a perpendicular buccal and palatal lines to the implant axis (width) [24] and through a parallel buccal and palatal lines to the implant axis at level of coronal part of the implant (length) (Figure 4). Keratinized buccal mucosa width was measured at 3 months in cases with immediate loading and at the removal of second-surgery suture in cases without immediate loading (< or > 2 mm of keratinized buccal mucosa width).

## 3. Results

Thirty-one zygomatic implants (13 Branemark System Zygoma ^®^ zygomatic implants (Nobel Biocare T.H, Sweden) and 18 Smooth IPX-Tilted System ^®^ zygomatic implants (Galimplant S.L, Sarria, Spain) were placed in 19 patients. The mean age was 61.7 years (54–73) and the gender proportion was 15.4% males and 84.6% females. Sinus membrane perforation during the surgery occurred in all cases. Immediate loading was performed in 41.9% of the total cases. The mean duration of follow-up was 20.1 months (range 12–41). Implant success rate was 100% and there were no biological complications.

Bone regeneration was performed around 16 zygomatic implants (Table 3). According to the ZAGA classification, 81.2% of the zygomatic implants with simultaneous bone regeneration were classified as ZAGA 1. Only one buccal regeneration failure among the total bone regenerations without implant failure was identified, due to presence of an infected fistula. The infected area was opened, the bone graft was curettage and retired, and irrigation with digluconate of chlorhexidine 0.12% was performed. The mean bone gain at 6 months showed that all successful cases obtained more than 2 mm of buccal and palatal bone width and length.

Soft tissue regeneration around zygomatic implants (Table 4) was performed in 15 implants. According to the ZAGA classification, 80% of the zygomatic implants with simultaneous soft tissue enhancement procedure were classified as ZAGA 1. No cases of soft tissue necrosis were recorded. The keratinized buccal mucosa width obtained was > 2 mm in all zygomatic implants.

## 4. Discussion

According to the literature, zygomatic implant therapy achieves survival rates between 92.3% and 100% [29]. Biological complications, such as maxillary sinusitis and soft tissue problems, have been reported [29].

Maxillary sinusitis is probably the most common biological complication with an incidence of up to 23.3% of all patients treated with zygomatic implants [30]. The coronal part of the implant is surrounded by an extremely atrophic alveolar bone crest, so marginal bone loss may easily result in an oroantral communication and consequent sinus infection [20,31,32,33]. As this study describes, transforming the atrophic bone crest related to the coronal part of the implant through bone or soft tissue enhancement techniques seems to be a logical strategy for avoiding such problems.

Chow et al. [23] described a simultaneous sinus lift for reducing oroantral communications and subsequent maxillary sinus infections in zygomatic implants. This technique preserved the integrity of the sinus mucosa and the implants were surrounded by bone during their intrasinusal trajectory. According to Chow et al. [23] no patients suffered maxillary sinusitis over 6 to 24 months of follow-up. In the present study, the implant length surrounded by bone was only centered around the coronal part in contrast with the implant completely covered in Chow’s technique. This postoperative bone-to-implant contact seems to be also enough to prevent maxillary sinusitis.

Hinze et al. [24] with Chow’s technique described an increase in peri-implant bone around the coronal part of the implant (buccal bone 1.4 ± 0.5 mm and palatal bone 4.3 ± 0.4 mm) at 6 months. The results of this study showed similar bone gained width (buccal bone 2.65 mm and palatal bone 3 mm) at 6 months. The new bone regeneration approaches suggest new bone formation around zygomatic implants at the coronal level, as with other published procedures from CBCT images [24].

Some zygomatic implants needed palatal bone regeneration because the coronal part of the implant had been placed in a palatal position with respect to the residual bone crest. This regeneration method was derived from palatal-positioned conventional implants in atrophic maxillae grade IV [6]. Palatal positioned implants are anchored in the palatal cortical bone with 2 to 5 exposed implant threads in their palatal surface. Peñarrocha-Diago et al. [34] placed 330 palatal positioned implants with simultaneous palatal bone regeneration in 69 severely resorbed edentulous maxillae that were rehabilitated with total fixed prostheses. The success rate of palatal positioned implants was 97.8% with a follow-up of 2 years and the peri-implant soft and bone tissue showed same values as well-centered implants in non-atrophic zones [35]. The palatal regeneration technique was successful in the 7 zygomatic implants in which it was used, with a gained bone width and length of 3 and 6.5 mm, respectively.

Some zygomatic implants needed buccal bone regeneration because the coronal part of the implant had a bone dehiscence, and more than the half of the implant was inside the bone alveolar crest. Wessing et al. [36] described that conventional implant survival was similar in simultaneous and deferred implant placements in guided bone regeneration with particulate graft materials and resorbable collagen membranes. Jung et al. [37] showed buccal bone width gain between 2–3 mm through CBCT measurements, such as the results of this study (2.65 mm). 

The peri-implant mucosa around zygomatic implants may also present complications such as buccal dehiscence, with the exposure of implant threads in the oral cavity. Thus, the soft tissue enhancement technique may prove crucial in extramaxillary zygomatic implants because the coronal part stays lateral of the alveolar bone crest [15,18,19].

The extramaxillary approaches have yielded high success rates, with the prevention of maxillary sinusitis. Aparicio et al. [38] reported a 100% survival rate and no cases of maxillary sinusitis in 20 patients with 63 extramaxillary zygomatic implants with a follow-up between 12 and 24 months. The extrasinus technique was introduced by the authors to avoid sinus complications and bulky prostheses in the presence of pronounced maxillary wall concavities. Implant paths started with a “tunnel” osteotomy thorough the alveolar remain from the palatal side of the crest. Soft tissue problems were not observed, nor even imagined the possibility to occur, and subsequently they were not reported. However, the recession of buccal mucosa may be presented, and infective and aesthetic problems may be produced when treating not so concave or more atrophied anatomies [18,19].

As a solution to these soft tissue complications, Guennal et al. [25] dissected the buccal fat pad in 25 patients treated with 62 zygomatic implants and no buccal recessions were produced. Aparicio et al. [26] proposed the ZAGA “Scarf Graft” to gain width of keratinized mucosa around zygomatic implants through pediculate connective tissue flap. In the present study, pediculate connective tissue flaps (ZAGA Scarf graft [26]) or free connective tissue grafts were executed. More than 2 mm of keratinized buccal mucosa were present in all zygomatic implants. This minimum value of 2 mm of keratinized buccal mucosa seems to be preventive with respect to the marginal bone loss in conventional implants [21]. Soft tissue grafting procedures showed successful results obtained in conventional implants regarding to less rate of bleeding and marginal bone loss [39].

The potential advantage of these bone and soft tissue enhancement methods is to optimize peri-implant tissues surrounding of the coronal part of the zygomatic implant. This allows regeneration of the atrophic maxillary bone crest and the prevention of biological complications around zygomatic implants.

There are some limitations to this study: The study design was a retrospective case series (low scientific level) with a short follow-up and a small sample, the immediate loading protocol was sometimes subjected to the patient’s demand and not only to the insertion torque value, and the measurement of the keratinized buccal mucosa was another aspect to improve because it was measured as a dichotomous value (major or minor than 2 mm). Therefore, prospective-controlled studies with sample size calculations, and longer follow-up times are necessary to prove if regenerative bone and soft tissue procedures reduce the occurrence of biological complications.

## 5. Conclusions

Within the limitations of the present study, bone regeneration and soft tissue enhancement techniques were useful to establish favorable conditions of the peri-implant tissues around zygomatic implants. This could prevent biological complications such as maxillary sinusitis and soft tissue recessions. Prospective and randomized controlled trials with a longer follow-up period are advisable.

## Figures and Tables

**Table 1:** Patient selection criteria.

Inclusion Criteria	Exclusion Criteria
Patient with indication for zygomatic implant treatment for atrophic maxillae (Cawood–Howell grade V and VI).	Cases with less than 12 months of follow-up.
Zygomatic implants with simultaneous bone regeneration or enhancement of peri-implant soft tissue.	Incomplete medical history and incomplete radiographic examination.

**Table 2:** Indication criteria for bone regeneration or soft tissue enhancement techniques.

Indicated Technique	Implant Emergence	Alveolar Process State		Illustrated Scheme *
		Width and Length of Palatal Bone Crest	Width and Length of Buccal Bone Crest	
Palatal bone regeneration	Palatal emergence.	Non-existent	Preserved.	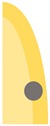
Palatal and buccal bone regeneration	Crestal emergence.	Non-existent or < 2 mm in both directions	Non-existent or < 2 mm in both directions.	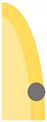
Buccal bone regeneration	Moderate buccal emergence with at least more than half of the implant diameter inside the alveolar bone crest.	Preserved.	Non-existent or < 2 mm in both directions.	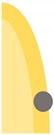
Soft tissue enhancement	Total buccal emergence or more than half of the implant diameter outside the alveolar bone crest.	Preserved.	Non-existent.	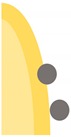

*: this scheme represents an occlusal view of the implant emergences in relation with the edentulous maxillae. The gray points are the emergence of zygomatic implants.

**Table 3:** Data referred to number of patients, number of zygomatic implants, ZAGA classification, immediate loading, successful regeneration technique, bone gain at the 6 months and biological complication at the implant level according to the type of bone regeneration technique.

Type of Bone Regeneration Technique	No. P	No. ZI	ZAGA Classification (%)					Immediate Loading. (%)	Successful Regeneration Technique (%)	Bone Gain at the 6 Months (mm)				Biological Complications (%)
			0	1	2	3	4			Width		Length		
										P	B	P	B	
**Buccal**	4	5	20	80	0	0	0	20	80	-	3	-	9.2	0
**Palatal**	4	7	14.3	71.4	14.3	0	0	28.6	100	3.7	-	5	-	0
**Buccal and Palatal**	2	4	0	100	0	0	0	50	100	2.3	2.3	8	7.4	0
**Total**	**10**	**16**	**12.5**	**81.2**	**6.2**	**0**	**0**	**31.25**	**93.75**	**3**	**2.65**	**6.5**	**8.3**	**0**

No.P: number of patient. No.ZI: number of zygomatic implants. P: palatal. B: buccal.

**Table 4:** Data referred to number of patients, number of zygomatic implants, ZAGA classification, immediate loading, successful enhancement technique, keratinized mucosa gain and biological complication at the implant level according to the type of soft tissue enhancement technique.

Type of Soft Tissue Enhancement Technique	No. P	No. ZI	ZAGA Classification (%)					Immediate Loading (%)	Successful Enhancement Technique (%)	Buccal Keratinized Mucosa Gain > 2 mm (mm)	Biological Complications (%)
			0	1	2	3	4				
**Pediculate Connective Tissue Graft**	**8**	**11**	**0**	**90.9**	**9.1**	0	0	72.7	100	100	0
**Free Connective Tissue Graft**	1	4	50	50	0	0	0	0	100	100	0
**Total**	**9**	**15**	**13.1**	**80**	**6.7**	**0**	**0**	**53.3**	**100**	**100**	**0**

No.P: number of patient. No.ZI: number of zygomatic implants.
